# Lack of basic and luxury goods and health-related dysfunction in older persons; Findings from the longitudinal SMILE study

**DOI:** 10.1186/1471-2458-8-242

**Published:** 2008-07-17

**Authors:** Daniëlle AI Groffen, Hans Bosma, Marjan van den Akker, Gertrudis IJM Kempen, Jacques TM van Eijk

**Affiliations:** 1 School for Public Health and Primary Care (CAPHRI), Department of Social Medicine, Maastricht University, PO Box 616, 6200 MD Maastricht, the Netherlands; 2 School for Public Health and Primary Care (CAPHRI), Department of General Practice, Maastricht University, the Netherlands; 3 School for Public Health and Primary Care (CAPHRI), Department of Health Care and Nursing Science, Maastricht University, the Netherlands

## Abstract

**Background:**

More so than the traditional socioeconomic indicators, such as education and income, wealth reflects the accumulation of resources and makes socioeconomic ranking manifest and explicitly visible to the outside world. While the lack of basic goods, such as a refrigerator, may affect health directly, via biological pathways, the lack of luxury goods, such as an LCD television, may affect health indirectly through psychosocial mechanisms. We set out to examine, firstly, the relevance of both basic and luxury goods in explaining health-related dysfunction in older persons, and, secondly, the extent to which these associations are independent of traditional socioeconomic indicators.

**Methods:**

Cross-sectional and longitudinal data from 2067 men and women aged 55 years and older who participated in the Study on Medical Information and Lifestyles Eindhoven (SMILE) were gathered. Logistic regression analyses were used to study the relation between a lack of basic and luxury goods and health-related function, assessed with two sub-domains of the SF-36.

**Results:**

The lack of basic goods was closely related to incident physical (OR = 2.32) and mental (OR = 2.12) dysfunction, even when the traditional measures of socioeconomic status, i.e. education or income, were taken into account. Cross-sectional analyses, in which basic and luxury goods were compared, showed that the lack of basic goods was strongly associated with mental dysfunction. Lack of luxury goods was, however, not related to dysfunction.

**Conclusion:**

Even in a relatively wealthy country like the Netherlands, the lack of certain basic goods is not uncommon. More importantly, lack of basic goods, as an indicator of wealth, was strongly related to health-related dysfunction also when traditional measures of socioeconomic status were taken into account. In contrast, no effects of luxury goods on physical or mental dysfunction were found. Future longitudinal research is necessary to clarify the precise mechanisms underlying these effects.

## Background

Adverse socioeconomic circumstances affect probabilities of good health and risks of disease [1,2]. Less clear is whether this also holds for older populations of whom many are retired and not in the paid labour force anymore [3,4]. In such populations, wealth may be a more valid indicator of socioeconomic ranking than the traditional indicators of socio-economic status, such as education and income. Wealth refers to the individual's or household's total financial resources amassed over the course of life [5]. Hence, the cumulative character of wealth might be important, particularly among older persons because of such life course effects. Furthermore, through being wealthy or not, socioeconomic ranking becomes explicitly visible to the outside world, more than through variations in educational and income level. For example, possession of goods, such as a car and a house, may be an explicit projection of how wealthy a household is.

Although differently measured across studies [5-8], wealth can be measured by the possession or lack of basic (e.g. refrigerator) and luxury goods (e.g. LCD television) [7]. Lack of basic goods may have direct biological effects on health [7,9]. For example, the lack of a refrigerator has been shown to increase the risk of stomach cancer [10]. The lack of such basic goods may, further, be related to the lack of sufficient or qualitatively good food and poor housing conditions (e.g. cold and draught), factors that more generally have their sources in the material world [9]. According to some scholars, this neo-material hypothesis is the most important explanation of health differences [9,11].

For the lack of luxury goods in a household, however, it is much more difficult to imagine direct biological effects. The visibility of socioeconomic ranking might be particularly important for the luxury items. The ownership of a large LCD television (preferably visible through the windows) or two cars (preferably both in front of the house) may be an expression of conspicuous consumption and be considered as an outward-directed symbol of status and prestige. The resulting psychosocial comparisons with others (having less or more of such goods) emphasise the potential relevance of psychosocial explanations of socioeconomic differences in health, rather than material explanations alone [7,12-14].

While wealth might be particularly important for an older population because of its cumulative character, the effects of specific measures of wealth on health-related functioning, independent of education and income, have not been widely studied among older persons [5,15]. Using cross-sectional and longitudinal data from the SMILE study, we set out to examine, firstly, the links between the possession of both basic and luxury goods and both mental and physical dysfunction in older Dutch men and women, and, secondly, the extent to which these links are independent of more traditional socioeconomic indicators, such as education and income.

## Methods

### Design

Data came from the longitudinal SMILE study (i.e. Study on Medical Information and Lifestyles Eindhoven), which started in November 2002 as a joint project of Maastricht University and the Eindhoven Corporation of Primary Health Care Centres. Eindhoven is a moderate city of approximately 200,000 inhabitants in the Southern part of The Netherlands. General practitioner's registers and annual postal questionnaires were used to collect data on health, lifestyles, and health care use. SMILE is a dynamic cohort, meaning that new participants will enter the population sample when they either reach the age of inclusion or when they are enrolled as a new patient in one of the participating centres and give informed consent. Responders may leave the population either through leaving the participating centres or due to death or drop-out [16]. Persons aged 55 years and older are considered as a separate population within SMILE [17].

### Study population

The present study uses data that were collected in May 2004 and May 2007. In May 2004, 11,180 persons of 55 years or older were sent a self-administered questionnaire, of whom 5,109 (46%) responded. Forty-two percent (N = 2,131) were followed up until May 2007. Data on educational level was extracted from the May 2003 questionnaire. After exclusion of persons that have missing scores on variables of interest, the main analyses consisted of 2,067 persons (989 men and 1078 women; mean age = 67.6, SD = 7.5).

### Ethical review

Written informed consent was asked when a patient registered in one of the participating health care centers. Privacy regulations are in agreement with the Dutch legislation. The medical ethical committee of the Maastricht Academic Hospital has approved of the study protocol of the SMILE study. Furthermore, the study was registered at the Dutch Data Protection Authority [16].

### Measures

#### Health-related function

Information about mental and physical dysfunction was derived from the Dutch version of the MOS SF36 [18,19], assessed in May 2004 and May 2007. The SF36 is a short-form health survey with 36 questions, clustered in eight subscales related to functional health and well-being. The eight scales can be recoded in two distinct higher-order components, i.e. physical and mental function [19,20]. For the purpose of this study, physical and mental dysfunction was defined as having a score below the 10th percentile (scores of ≤ 30 and ≤ 36 out of a range from 10 to 75, respectively). Furthermore, persons that have more than 50% missing scores on physical or mental function were excluded from the analyses [19].

#### Basic and luxury goods

Basic goods, measured in May 2004, included a freezer, oven, washing machine, refrigerator, telephone, car and own house [7,17,21]. Intentionally, three categories were created in such a way that each group contained approximately a third of the sample at baseline: the possession of 0 to 4 basic goods, possession of 5 or 6 basic goods, and possession of all 7 basic goods. Luxury goods have been measured in May 2007 and included a dishwasher, (tumble) dryer, solarium, microwave oven, DVD-player, DVD-recorder, video camera, PC (desktop), laptop, mp3-player, internet connection, plasma/LCD television, cell-phone, caravan/trailer, second house, musical instrument, second car, navigation system in car, digital television, game console, and a digital photo camera [7]. Again, three categories were created: the possession of 0 to 5 luxury goods; possession of 6 to 10 luxury goods, and possession of 11 to 21 luxury goods.

#### Covariates

Covariates were age, gender, educational level, and prevalent severe and less severe disease. Education was measured in May 2003, using a seven-point scale. Three categories were then created in such a way that each group contained approximately a third of the sample: primary school only (lowest); lower vocational education and intermediate general education (middle); intermediate vocational education, higher general education, higher vocational education, and university (highest). Income and financial problems were also separately controlled for, instead of educational level, but as findings were very similar, despite income having more missing scores, only findings for the educational control are presented. Respondents were further asked whether or not they had any of the severe (COPD, heart disease, bowel disease, liver disease, kidney disease, diabetes, cancer, epilepsy, and stroke) and less severe (migraine, joints, rheumatoid arthritis, arthrosis, back, injury, and other) diseases [22]. Presence of diseases was measured in May 2004 and May 2007.

### Statistical analyses

Chi-square tests, based on cross-tabulations, were computed to examine whether there were gender, age, disease status, and physical and mental function differences in the possession of basic or luxury goods. The associations of educational level with the possession of basic and luxury goods and with dysfunction were analysed as well. Multiple logistic regression models were fitted to examine whether lack of basic or luxury goods were associated with physical and mental dysfunction (measured in 2007). Possession of all of the selected basic goods or the highest category of luxury goods was the reference category. The first model was adjusted for age and sex. The second model comprised age and gender and was simultaneously adjusted for both the possession of luxury and basic goods. In the third model, odds ratios were further adjusted for educational level. The final model additionally controlled for the presence of severe (0–9) and less severe (0–7) diseases (measured in 2007). For the basic goods, longitudinal analyses were also done to study the relation with incident dysfunction between 2004 and 2007 (N = 124). Prevalent dysfunction at the 2004 baseline was excluded (N = 150). A similar sequence of models as described above was used, adjusting for severe and less severe diseases at baseline (measured in 2004). Finally, exploratory analyses were performed for different subgroups, i.e. younger (55–65) and older (>65) persons, male and female, persons with and without chronic diseases and persons with high or low education. All statistical analyses were performed using SPSS 14.0.1.

## Results

Older persons and women more often reported a relative lack of basic and luxury goods (not tabulated). For example, persons aged 75 or older were more likely to report owning four or less of the basic goods (20%) than persons between 55 and 64 years of age (5%). Furthermore, women were significantly more likely to report owning four or less of the basic goods (11%) than men (7%). Similar associations were found for the lack of luxury goods. Seventy percent of persons aged 75 years and older reported owning five or less of the luxury goods, while only 20% of persons between 55 and 65 years of age did so. Furthermore, only 4% of the oldest age group reported owning 11 to 21 of the luxury goods, while this was reported by 32% of the youngest age group. Moreover, men were more likely to report owning 11 to 21 of the luxury goods (22% versus 15%), and were less likely to report having five or less of the luxury goods (30% versus 45%) compared with women.

Table 1 presents the associations between the lack of basic and luxury goods and the prevalence of less severe and more severe diseases and physical and mental dysfunction. Persons owning four or less of the basic goods were more likely to suffer from severe diseases (31% versus 24%) and physical and mental dysfunction (19% versus 6%) than persons owning all of the selected basic goods. The lack of luxury goods was also related to the prevalence of severe diseases and physical and mental dysfunction, although associations were somewhat weaker.

**Table 1:** Percentages of participants with diseases and with relative poor physical or mental function^123^

	**Total**	**≥ 1 less severe disease**	**≥ 1 severe disease**	**Physical dysfunction**	**Mental dysfunction**
	*N = 2858*^4^	*N = 1139*	*N = 769*	*N = 262*	N = 239
**Basic goods**					
**≤ 4**	*N = 252*	42.1	***31.3***	***19.4***	***18.9***
**5–6**	*N = 1566*	40.4	***28.4***	***12.1***	***10.6***
**7**	*N = 1023*	39.3	***24.2***	***6.3***	***5.9***
**Luxury goods**					
**≤ 5**	N = 1081	39.3	***31.2***	***16.2***	***12.2***
**6–10**	N = 1232	40.7	***25.2***	***7.1***	***8.2***
**11–21**	N = 514	41.4	***23.5***	***7.2***	***7.0***

^1 ^Row percentages, for basic and luxury goods separately.

^2 ^Results in bold indicate a significant difference (X^2^; p < 0.05).

^3 ^Diseases were self-reported.

^4 ^Basic and luxury goods have different number of missing values.

Examination of the individual basic items (not tabulated) showed that not owning one's own house (52%), car (17%), or freezer (16%) were most often reported. Moreover, the lack of a car and own house were most strongly related to dysfunction. For example, 20% of persons not owning a car suffered from physical dysfunction compared with 7% of the car owners. Persons not owning a washing machine were more likely to report mental dysfunction (19% versus 9%) than those who possessed such a machine. Examination of the individual luxury items showed that it was particularly the lack of a personal computer, internet connection, and a mobile phone which were associated with dysfunction. For example, almost 15% of persons lacking an internet connection at home reported physical dysfunction, compared with only 7% of persons who had an internet connection available.

There was a significant positive association between the number of basic goods and the number of luxury goods (Spearman's R = 0.50, P < 0.001 using the continuous measures). Cross tabulations (not presented) showed that 84% of persons owning only four or less of the basic goods reported owning only five or less of the luxury goods, compared with only 18% of persons owning all of the basic goods.

Table 2 shows how education was associated with the possession of basic and luxury goods and health-related dysfunction. Twenty percent of persons with a low educational level reported owning only four or less of the basic goods, while only 4% of persons with a high educational level did so. Furthermore, 16% of persons with a low educational level reported poor physical and mental functioning, while only 7% in the group with a high educational level did so.

**Table 2:** The association of educational level with basic and luxury goods and health-related function^123^

**Education**	**Basic goods**			**Luxury goods**			**Physical function**		**Mental function**	
	**≤ 4 N = 195**	**5–6 N = 1280**	**7 N = 839**	**≤ 5 N = 868**	**6–10 N = 1015**	**11–21 N = 417**	**Healthy N = 1871**	**Poor N = 208**	**Healthy N = 1893**	**Poor N = 185**

Low	19.8	67.4	12.8	65.2	28.7	6.0	84.2	15.8	84.2	15.8
Middle	8.5	60.8	30.8	41.0	42.9	16.1	89.1	10.9	91.4	8.6
High	3.9	45.7	50.5	24.0	51.3	24.7	92.7	7.3	93.1	6.9

^1 ^Row percentages.

^2 ^Results in bold indicate a significant difference (X^2^; p < 0.05).

^3 ^Basic and luxury goods have different number of missing values.

Table 3 shows that there was a substantial association between the lack of basic goods and mental dysfunction. Persons with four or less basic goods had more than twice the risk of mental dysfunction (OR = 2.56) of persons owning all of the selected basic goods. The significance of these associations even held after additional adjustment for luxury goods, educational level and severe and less severe diseases (OR = 2.13). The lack of basic goods was also related to physical dysfunction (OR = 1.91). However, the odds ratios became non-significant, when controlled for educational level and diseases. In contrast, the lack of luxury goods was not related to either mental or physical dysfunction. The unadjusted significant association between the relative lack of luxury goods and dysfunction, as shown in Table 1, disappeared when controlled for age and sex. Those without luxury goods being older, in particular, explained 85% of the corresponding unadjusted odds ratio of 2.59 (95% CI: 1.66–4.04).

**Table 3:** Adjusted Odds Ratios (OR) of physical and mental dysfunction by basic and luxury goods

	**Model 1^1 ^**OR (95% CI)	**Model 2^2 ^**OR (95% CI)	**Model 3^3 ^**OR (95% CI)	**Model 4^4 ^**OR (95% CI)
**Basic goods**	**Physical dysfunction (N = 2067)**			
≤ 4	1.91 (1.10–3.33)	1.65 (0.93–2.93)	1.49 (0.83–2.68)	1.28 (0.68–2.42)
5–6	1.62 (1.15–2.29)	1.51 (1.06–2.15)	1.42 (0.99–2.04)	1.41 (0.96–2.08)
7	1.00	1.00	1.00	1.00
**Luxury goods**				
≤ 5	1.54 (0.94–2.52)	1.32 (0.79–2.19)	1.25 (0.75–2.09)	1.11 (0.64–1.92)
6–10	0.88 (0.55–1.42)	0.82 (0.51–1.32)	0.81 (0.50–1.31)	0.77 (0.46–1.28)
11–21	1.00	1.00	1.00	1.00
**Basic goods**	**Mental dysfunction (N = 2067)**			
≤ 4	2.56 (1.48–4.34)	2.47 (1.39–4.41)	2.18 (1.21–3.93)	2.13 (1.17–3.84)
5–6	1.53 (1.08–2.18)	1.51 (1.05–2.17)	1.39 (0.96–2.02)	1.37 (0.95–1.99)
7	1.00	1.00	1.00	1.00
**Luxury goods**				
≤ 5	1.33 (0.82–2.16)	1.04 (0.62–1.74)	0.97 (0.58–1.62)	0.91 (0.54–1.53)
6–10	1.02 (0.65–1.60)	0.91 (0.58–1.44)	0.90 (0.57–1.43)	0.88 (0.56–1.40)
11–21	1.00	1.00	1.00	1.00

^1 ^Model 1 is adjusted for age and gender.

^2 ^Model 2 is adjusted for age, gender and simultaneously for luxury goods or basic goods.

^3 ^Model 3 is additionally adjusted for education.

^4 ^Model 4 is additionally adjusted for severe and less severe diseases.

Longitudinal analyses showed that the lack of basic goods was related to both incident mental and physical dysfunction, even after additional adjustment for gender, age, luxury goods, educational level, and severe and less severe disease (OR = 2.32 for physical functioning and OR = 2.12 for mental functioning) (Table 4).

**Table 4:** Odds ratios (OR) of the incidence of physical and mental dysfunction by basic goods^1^

	**Model 1^2 ^**OR (95% CI)	**Model 2^3 ^**OR (95% CI)	**Model 3^4 ^**OR (95% CI)	**Model 4^5 ^**OR (95% CI)
**Basic goods**	**Physical functioning (n = 1769)**			
≤ 4	2.56 (1.17–5.60)	2.52 (1.11–5.70)	2.63 (1.14–6.07)	2.32 (1.00–5.38)
5–6	1.73 (1.04–2.89)	1.71 (1.01–2.90)	1.76 (1.03–3.00)	1.70 (0.99–2.90)
7	1.00	1.00	1.00	1.00
**Basic goods**	**Mental functioning (n = 1761)**			
≤ 4	2.50 (1.26–4.94)	2.52 (1.23–5.16)	2.30 (1.11–4.79)	2.12 (1.01–4.73)
5–6	1.35 (0.87–2.11)	1.36 (0.86–2.15)	1.29 (0.81–2.05)	1.26 (0.79–2.01)
7	1.00	1.00	1.00	1.00

^1 ^Prevalent dysfunction cases in 2004 are excluded from the analyses.

^2 ^Model 1 is adjusted for age and gender.

^3 ^Model 2 is additionally adjusted for luxury goods.

^4 ^Model 3 is additionally adjusted for education.

^5 ^Model 4 is additionally adjusted for severe and less severe diseases.

Effects were similar in men and women, for the younger (≤ 65) and older (>65) age groups, for diseased and non-diseased persons, and for both the lower and higher educated persons, as the respective interaction terms were not significant in our analyses. Furthermore, when using linear regression analyses with mental and physical function as continuous variables, similar associations were found.

## Discussion

In this group of older Dutch men and women, a lack of basic goods was not uncommon. Moreover, this lack of basic goods, as indicator of wealth, turned out to be a good predictor of both incident physical and mental dysfunction, even when traditional measures of socio-economic status, i.e. education and income, were taken into account. No significant associations were found between the lack of luxury goods and dysfunction. All association were independent of the prevalence of severe and less severe diseases.

Given that basic goods are indicators of wealth [5-8], our results suggest that for an older population wealth might be an additional or even more appropriate predictor of health-related dysfunction than the traditional measures of SES, i.e. education and income. This may be due to its cumulative character, indicating economic advantage and disadvantage amassed over the course of life. Wealth may, further, buffer the effects of lost or temporarily low income [5,15].

The lack of basic goods having more impact on health-related dysfunction than the lack of luxury goods is consistent with the view that material factors are important determinants of health. Living in poor material conditions or with a lack of resources may have a direct, biological, effect on health [9]. Furthermore, the lack of a car makes shopping for (healthy) food and access to health care services much more difficult and the ownership of a house may, on average, be associated with a better housing conditions [23].

However, psychological or psychosocial pathways cannot be excluded either. The lack of a car, refrigerator, or oven is visible for neighbours, friends, and acquaintances. The negative social comparison resulting from this apparent visibility might have adverse effects on self-esteem and subjective prestige, pride, and status through which eventually both mental and physical dysfunction might become compromised as well [12,14,24]. The possibility that psychological pathways might contribute is also substantiated by recent experiences with Dutch food banks. These are increasingly being moved to the suburbs, where their customers report less shame and other psychosocial problems, given that their visits to these more secluded environments are less likely to be noticed [25]. Perhaps shame may even be more prominent when lacking sufficient food or a refrigerator than a second house or a DVD recorder.

Why then are there no effects of a lack of luxury goods, where visibility and corresponding psychosocial pathways were hypothesised to have particular relevance? Our data suggest that the possession of a large LCD television or a second car does not make older persons healthier and happier compared with their neighbours who do not have such items. Pikhart and colleagues (2003), however, found that luxury items remained strongly associated with self-rated health even in fully adjusted models. It should, however, be noticed that this finding only held for a, on average younger (18+) Hungarian population. Similar to our study, luxury goods were only studied using cross-sectional data. Future longitudinal research is necessary to clarify the relations. As mentioned above, visible status, social comparisons and related psychosocial mechanisms [26] might also, and perhaps particularly, hold for explaining the adverse health outcomes of lacking basic goods.

### Methodological considerations

Several methodological limitations may affect the interpretation of the results of our study. Firstly, the selection of items is cultural [27] and time-dependent. The individual basic and luxury good items were chosen on the basis of recent reports [7,21]. Furthermore, as we state in our results section, certain items have more predictive power than others. However, before reducing the scales to the items that are predictive for dysfunction, more research is needed, also with other health outcomes. Further development and validation of measurement instruments to assess the possession of both basic and luxury goods is recommended. Moreover, more attention should be paid to additional measures of poor material circumstances, such as poor physical housing and working conditions (e.g. dampness, mould, and cold in the house and lifting heavy loads at work) [28,29].

Secondly, our study relied solely on self-reports which might have introduced measurement error for the dysfunction measures [30]. Individuals with a general tendency towards negative perceptions of material well-being may also over-report symptoms of health-related dysfunction (i.e. negative affectivity) [31]. This may have led to an overestimation of the presented association. However, by excluding prevalent dysfunction cases from the longitudinal analyses, persons with negative affectivity [32,33] have also been excluded. Overlap between the physical and mental functioning component of the SF-36 outcome measure might also have distorted our results [34]. However, these measures were only weakly correlated (Pearson's R = 0.168, p < 0.001) within our study population. Furthermore, poor people may have underreported their poverty. Out of shame, people may not want to admit not being able to afford certain (basic) items. This bias may have led to an underestimation of the presented associations.

Thirdly, the question remains why the longitudinal association between basic goods and physical dysfunction is stronger than the corresponding cross-sectional association. Except that longitudinal analyses are generally considered superior because of the exclusion of reverse causation, we could not come to an answer to this question. More research is recommended to disentangle possible underlying mechanisms.

Finally, our research may be limited by potential selection biases. Older persons living in nursing homes were not included which restricts the generalisability of our findings. The most disadvantaged older persons may be underrepresented in our research, because of premature mortality (the 'wealthy survivor' effect). Response analyses showed that men and persons from the youngest (<65) age groups were more likely to respond. Furthermore, attrition (between 2004 and 2007, 33% were lost to follow-up) was higher for persons that were lower educated and had worse scores on physical and mental function scores at the 2004 baseline (P < 0.001). Missing values analyses revealed that persons with missing values on mental and/or physical function scales were significantly more likely to be lower educated, have fewer basic and luxury goods and have worse physical and mental function scores at the 2004 baseline. Similarly, persons with missing values on basic or luxury good items were more likely to report poor mental and physical function. This pattern of selective response and attrition may have led to an underestimation of the reported associations, although attrition's effect may be more disturbing for descriptive results than for measures of (longitudinal) association [35].

## Conclusion

Even in a relatively wealthy country like the Netherlands, the lack of certain basic goods is not uncommon. More importantly, lack of basic goods, as indicator of wealth, was strongly related to health-related dysfunction, also when taking into account traditional measures of socioeconomic status. In contrast, no effects of luxury goods on physical or mental dysfunction were found. Future longitudinal research is necessary to clarify the precise mechanisms underlying the effects and -particularly in older persons- to explore strategies to intervene upon the adverse effects of material deprivation.

## Competing interests

The authors declare that they have no competing interests.

## Authors' contributions

DAIG performed the statistical analyses and drafted the manuscript. HB formulated the hypotheses. MvdA was responsible for the design of the SMILE study. All authors (HB,MvdA,GIJMK,JTMvE) edited and approved the final manuscript.

## Pre-publication history

The pre-publication history for this paper can be accessed here:


